# Antimicrobial use prior as a risk factor for developing extended-spectrum beta-lactamse-producing Klebsiella spp. in South Brazil

**DOI:** 10.1186/1753-6561-5-S6-P138

**Published:** 2011-06-29

**Authors:** T Ostroski, C Ito, C Busato, MD da Rocha, C da Silva, V Bian, L Bail

**Affiliations:** 1 Departamento de Medicina, Universidade Estadual de Ponta Grossa, Ponta Grossa, Brazil; 2 Análises Clínicas e Toxicológicas, Universidade Estadual de Ponta Grossa, Ponta Grossa, Brazil; 3 Departamento de Saúde Pública, Universidade Estadual de Ponta Grossa, Ponta Grossa, Brazil; 4 Intensive Care Unit, Santa Casa de Misericórdia, Ponta Grossa, Brazil; 5 Pharmacy, Santa Casa de Misericórdia, Ponta Grossa, Brazil

## Introduction / objectives

Extended-spectrum beta-lactamase-producing (ESBL) Klebsiella spp. is a problematic nosocomical pathogen around the world. In the present study, we aimed to evaluate the previous use of antibiotics as a risk factor for isolation of ESBL Klebsiella spp.

## Methods

In aÂ retrospective case control study between June 2009 and June 2010 at Santa Casa de Misericórdia Hospital, city of Ponta Grossa, south Brazil, 61 *Klebsiella* spp. (29 ESBL and 32 controls non-ESBL-producing isolates) were enrolled. ESBL were screened by disk diffusion method and double disk approximation method, according to CLSI. Prior use of antibiotic was analyzed in electronic medical records. The antibiotic consumption (DDDs – defined daily doses) was tested using the X^2^ test (*p* <0.05).

## Results

The DDDs of prior use and full use of cephalosporines, fluoroquinolones, e metronidazole in ESBL and non-ESBL groups were (122.02/145.77; 8.78/69.07), (15.66/22; 0.01/26.2), (47.33/47.66; 3/28) respectively. Prior use of cephalosporines, fluoroquinolones, and metronidazole was higher in ESBL-*Klebsiella* spp. than non-ESBL-*Klebsiella* spp. (p<0,001). CarbapenemsÂ were not used by the control group.

## Conclusion

Theprior use of broad-spectrum cephalosporins, fluoroquinolones and metronidazole is an important risk factor for acquisition of ESBL producing Klebsiella spp.

## Disclosure of interest

None declared.

